# ﻿Two new genera of grasshoppers (Orthoptera, Acrididae, Melanoplinae) from Baja California, Mexico, with a regional key to the genera of Melanoplinae

**DOI:** 10.3897/zookeys.1238.147762

**Published:** 2025-05-13

**Authors:** JoVonn G. Hill

**Affiliations:** 1 405 East Garrard Road Mississippi Entomological Museum, Department of Agricultural Science and Plant Protection, Mississippi State University, Starkville, MS 39759, USA Mississippi State University Starkville United States of America

**Keywords:** *Bajatettix*, Cape Region, identification key, *Ozmacris*

## Abstract

Baja California, a 1,300 km long peninsula, exhibits considerable ecological diversity, encompassing coastal chaparral, coniferous forests, low desert scrub, and tropical deciduous forests. The region’s ecological complexity reflects its biogeographic history, marked by separation from mainland Mexico 5.5 million years ago. Survey efforts have documented an impressive 4,000 plants, and insect efforts have targeted bees, weevils, ants, and blow flies. Grasshoppers, in contrast, remain underexplored. The present study expands on expeditions from the 1970s to 2010s that focused on the peninsula’s Orthoptera. Two new genera are established—*Bajatettix* and *Ozmacris*—and a key to the genera of the Melanoplinae of the peninsula is provided. This study highlights the importance of understanding Baja California’s grasshopper diversity to support conservation initiatives and future ecological studies.

## ﻿Introduction

Baja California is a land of striking contrasts, characterized by diverse biological communities, such as coastal chaparral, coniferous forests, low desert scrub, and tropical deciduous forests, each supporting distinct ecological communities that contribute to the region’s remarkable biodiversity. This 1,300 km long peninsula extends southward from the southern border of California, covering 143,390 km^2^ and featuring 3,000 km of coastline, the Sierra Juárez and Sierra San Pedro Mártir mountain ranges, and more than 100 islands. The region’s complex biogeographic history and varied landscapes have fostered high levels of endemism. About 5.5 million years ago, the peninsula separated from mainland Mexico, forming the Gulf of California. Subsequent sea level changes further isolated portions of the peninsula, creating areas of endemism (Murphy 1983; Riddle et al. 2000). Today, the flora of Baja California includes over 4,000 plant taxa, about 30% of which are found exclusively in the states of Baja California and Baja California Sur (Baja Flora 2024).

The insect fauna of the Baja California peninsula has become increasingly well-documented in recent decades. Notably contributions include documenting 728 bees (De Pedro et al. 2024), 213 weevils (Hernández et al. 2024), 170 ants (Johnson and Ward 2002), and 16 blow flies (Stotelmyre 2024). These efforts have provided crucial understanding of the region’s biodiversity, which lays the foundation for research in other disciplines, such as conservation and ecology. However, grasshopper fauna remains poorly known.

Between the 1970s and 2010s, expeditions led by David Weissman, David Lightfoot, and Robert Love, specifically targeted Orthoptera with the goal of thoroughly documenting fauna of the peninsula. The extensive collections from these expeditions were distributed to other specialists for identification. In January 2024, by a somewhat circuitous route, the acridid specimens reached me. In this lot was the first known male of *Barytettixpeninsulae* (Scudder, 1897), which examination of this specimen revealed to not belong to *Barytettix* but represented an undescribed genus. Additionally, seven specimens could not be assigned to any existing genus. These discoveries prompted the descriptions presented herein.

Here, I describe two new genera—*Bajatettix* and *Ozmacris*—and provide a key to Melanoplinae of the Baja California peninsula. This work aims to facilitate future research on the region’s grasshopper fauna and contribute to a more comprehensive understanding of Baja California’s biodiversity.

## ﻿Materials and methods

Specimens examined in this study were borrowed from the California Academy of Sciences (**CAS**), the Academy of Natural Sciences of Drexel University (**ANSP**), and the United States National Collection (**USNM**). All type specimens of newly described species are deposited in the Mississippi Entomological Museum (**MEM**). Nomenclature follows Cigliano et al. (2024). Specimens collected by the MEM have been databased in the Ecdysis database. Observation records were gathered from iNaturalist (https://www.inaturalist.org/home) by examining all the Melanoplinae records from the Baja peninsula.

Internal male genitalia, which are typically concealed within the terminalia, were either exposed upon pinning fresh specimens, or the specimen was relaxed by soaking in warm water, then the genital mass was either extruded or dissected and examined in a manner similar to Gurney and Brooks (1959). Terminology for external morphology and male genitalia follows Carbonell (2007) and Eades (2000). Habitus and internal genitalia images were produced using a Leica DFC 495 digital camera mounted on a Leica Z16 microscope with motorized z-stepping. Image stacks were merged using Leica Application Suite v. 4.1.0 with the Montage Module. Images were edited using Adobe Photoshop CS6 software. A green label stating “Measured by JGH” was added to the specimens measured in this study. Measurements were made with a Leica MZ 12.5 stereomicroscope with a reticule in the following ways:

- Body length — dorsally from the fastigium of vertex to the distal end of the genicular lobe of the hind femur in a parallel plane with the abdomen
- Pronotum length — dorsally, along the median carina
- Male cercus length — laterally, maximum measurement of the left cercus
- Male cercus basal width — laterally, along the point of attachment from the dorsal to ventral margin
- Male mid cercus width — laterally, at the mid-length of the left cercus
- Male cercus ventral branch length — laterally, from beginning of the fork to the apex
- Male cercus ventral branch apex width — laterally, along the distal end
- Male cercus dorsal branch length — Laterally, from beginning of the fork to the apex
- Male cercus dorsal branch apex width — Laterally, along the distal end
- Aedeagus Length — caudally from point where ventral portion of the sheath ends to apex of longest valve (dorsal or ventral)
- Dorsal valve apex width — the distance spanned by the pair dorsally at the apex where both valves terminate
- Dorsal valve middle width — dorsally the distance spanned by the pair dorsally at mid-length
- Dorsal valve basal width — dorsally the distance spanned by the pair dorsally at the point where the valves emerge from the sheath
- Ventral valve apex width — the distance spanned by the pair ventrally at their distal apex
- Ventral valve middle width — ventrally the distance spanned by the pair dorsally at mid-length
- Ventral valve basal width — dorsally the distance spanned by the pair dorsally at the point where the valves emerge from the sheath
- Female dorsal ovipositor valve — Laterally, from the base to the apex
- Female ventral ovipositor valve — Laterally, from the base to the apex

## ﻿Taxonomic account

### ﻿Key to the genera of Melanoplinae of Baja California

Works best with male specimens but will work with females as well.

**Table:** 

1	Prosternum with a protuberant spine (Fig. 1A)	2
–	Prosternum without a protuberant spine	**not Melanoplinae**
2	Mesosternum with lateral lobes as wide as long or wider than long (Fig. 1B)	**3 (Melanoplinae)**
–	Mesosternum with lateral lobes longer than wide (Fig. 1C)	**not Melanoplinae**
3	Tegmina absent (Fig. 2A, B); male cerci acutely triangular (Fig. 3G)	**4**
–	Tegmina present (Figs 2C–H, 4A, B); male cerci variable as in Fig. 3H–K, but not limited to those shapes	**5**
4	Body surface rugose and punctate; mesothorax and metathorax not completely covered with a broad dark band; smaller in overall size (Fig. 2A)	***Psilotettix***
–	Body surface generally smooth; mesothorax and metathorax covered with a broad dark band (Fig. 2B); larger in overall size (Fig. 2B)	***Bajatettix* gen. nov.**
5	Brachypterous; tegmina bicolored, black on the lower two-thirds and white above (Figs 2C, 3A); overall green bodied; entire distal end of hind femur black; male cerci toothed distally (Fig. 3H)	***Ozmacris* gen. nov.**
–	Brachypterous or macropterous; tegmina not bicolored (Figs 2D–H, 4A, B); body color variable; cerci not toothed (Fig. 3I–K)	**6**
6	Brachypterous (Fig. 2D, E); hind margin of the pronotum broadly emarginate (Fig. 3C) or notched (Fig. 3D)	**7**
–	Macropterous or brachypterous; hind margin of the pronotum not broadly emarginate or notched (Fig. 3E, F)	**8**
7	Hind margin of the pronotum notched medially (Fig. 3D); hind tibia with seven external spines (Fig. 5A); distal end of the hind femur gray or brown in the genicular area; male subgenital plate not strongly conical (Fig. 2D); male cerci triangular	***Oedomerus***
–	Hind margin of the pronotum emarginate (Fig. 3C); hind tibia with more than seven external spines (Fig. 5B, C); distal end of hind femur only black in the genicular area; male subgenital plate strongly conical (Figs 2E, 4C); male cerci downcurved, falcate, or broadly enlarged distally (Fig. 3J	***Barytettix***
8	Head and pronotum with a white stripe medially (Fig. 3E); body green or greenish-brown overall, with red, white, and black markings (Fig. 2F)	***Hesperotettix***
–	Head and pronotum without a white medial stripe; body not greenish or brown overall with red, white, and black markings	**9**
9	Body deep through metathorax (Fig. 2G); dorsum of head and pronotum with distinctive pattern (Fig. 3F); male subgenital plate with a distinctive tubercle (Fig. 4D); ventro-basal margin of hind femur with a distinctive wedge-like structure below (Fig. 5B), brachypterous or macropterous; male cerci cuneate or narrowly triangular (Fig. 3K)	***Aeoloplides***
–	Body not deep through metathorax (Figs 2H, 4A, B); dorsum of head and pronotum without a pattern as in Fig. 3F; male subgenital plate variable; hind femur ventro-basal margin of hind femora rounded (Fig. 5 A, C)	**10**
10	Body stout with thick hind femora (Fig. 2H); pronotum with lateral ridges present and prozona swollen; male cerci broad at the base and abruptly narrowed in the distal third (Fig. 3I)	***Oedaleonotus***
–	Body variable; pronotum without lateral ridges or swollen prozona; male cerci variable (Fig. 3A, B)	***Melanoplus***

Below, the new genus—*Bajatettix* (Fig. 6A)—is described based on seven specimens collected by R.E. Love at 15.7 miles south of La Ribera and *Barytettixpeninsulae* is moved to the new genus *Ozmacris* (Fig. 6B). Morphology of the male phallic complex used in descriptions is given in Fig. 7A–D.

**Figure 1.**
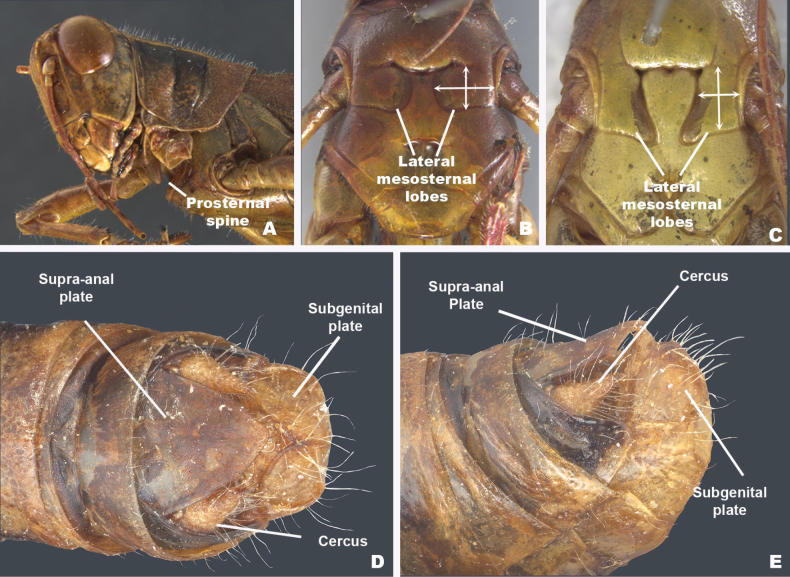
External characters used in the key **A***Melanoplus* prosternal spine **B**Melanoplinae thoracic metasternum **C**Cyrtacanthacridinae thoracic metasternum **D** dorsal view of male terminalia **E** lateral view of male terminalia.

#### ﻿ Bajatettix gen. nov.

Taxon classificationAnimaliaOrthopteraAcrididae

3D7A7700-8B28-56EF-8AC7-7343A46B3142

https://zoobank.org/C0B894E6-0B64-4D86-A3ED-84412923A974

**Figure 2.**
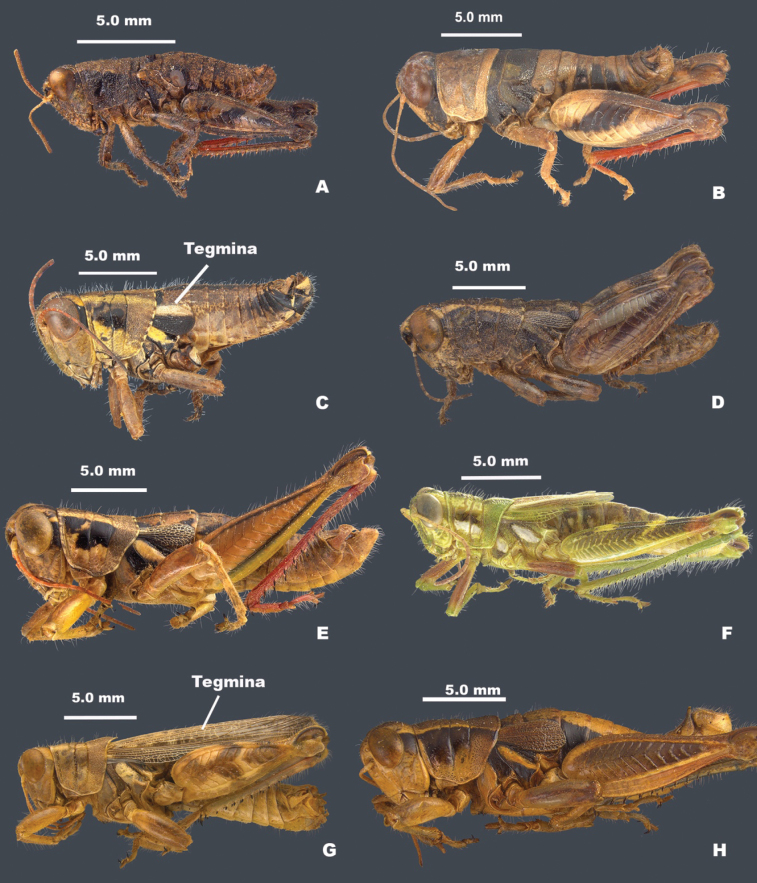
Habitus of melanopline genera of Baja California **A***Psilotettix***B***Bajatettix***C***Ozmacris* showing brachypterous bicolored tegmina **D***Oedomerus***E***Barytettix***F***Hesperotettix***G***Aeoloplides* showing macropterous tegmina **H***Oedaleonotus*.

**Figure 3.**
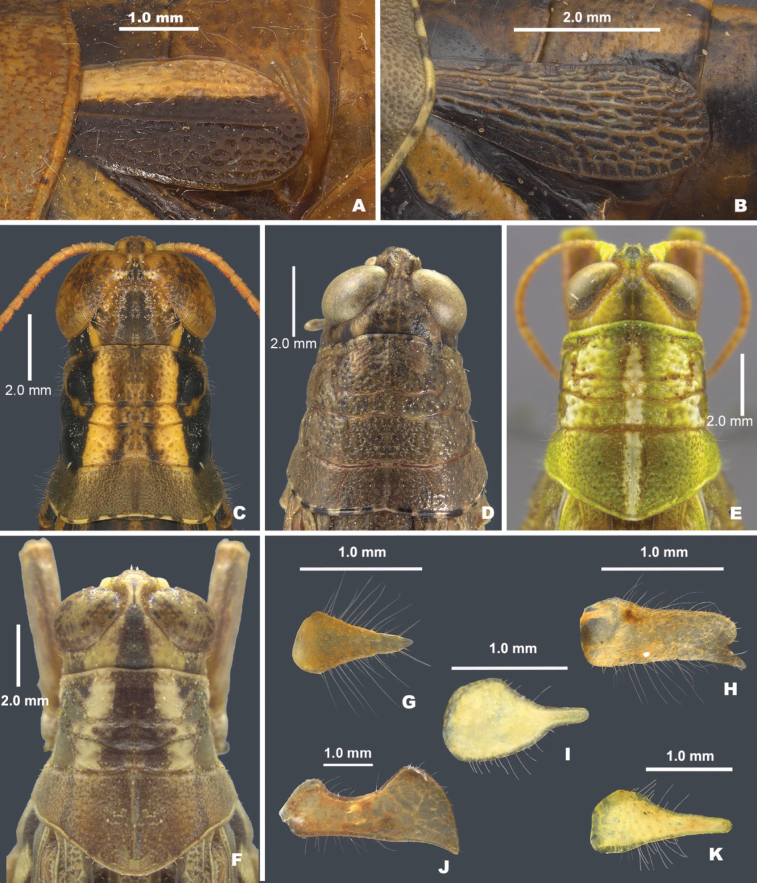
Anatomy of select melanoplines of Baja, California **A** tegmen of *Ozmacrispeninsulae***B** tegmen of *Barytettix***C** pronotum dorsum of *Barytettix***D** pronotum dorsum of *Oedomerus***E** pronotum dorsum of *Hesperotettix***F** pronotum dorsum of *Aeoloplides***G** cerci of *Bajatettix***H** cerci of *Ozmacris***I** cerci of *Oedaleonotus***J** cerci of *Barytettix***K** cerci of *Aeoloplides*.

Figs 2B , 3G , 6A , 8A–D , 9A , 10A–F , 11

##### Generic description.

***External morphology*.** Small (17.2–22.3 mm), apterous grasshoppers (Figs 2B, 6A). ***Head*** moderately sized and equal in width to the anterior edge of the prozona; vertex between the eyes much wider than the basal antennomere; fastigium broadly rounded being more pronounced dorsally than ventrally, with a dorsal shallow medial depression that is broad apically and narrow caudally. Eyes prominent, especially in males. Three ocelli present. Antennae filiform, usually with 20–23 flagellomeres in males, and 21–25 in females, nearly cylindrical, but slightly flattened dorso-ventrally, especially the distal two articles, equal in width throughout, except two basal articles. Clypeus trapezoidal with lateral sulci and a shallow medial notch on the ventral edge. **Thorax** with prosternal spine short, broad, and bluntly rounded distally. Pronotum convex in cross section, anterior margins broadly rounded, posterior margin truncate, medial carina cut by three sulci, lateral carinae absent and humeral margins rounded. Prozona punctate; lateral lobes with parallel lateral margins and the ventral margin sharply angled caudally. Metazona punctate throughout, with humeral margins rounded and in dorsal view, slightly diverging posteriorly. Median carina low, almost indistinct. Anterior, median, and posterior sulci are apparent, and all dissect the median carina and nearly reach the ventral margin of the lateral lobes. Lateral pronotal margins broadly rounded throughout. Interspace between mesosternal lobes quadrate, being as long as broad. Tegmina absent. Pro- and meso-thoracic legs not robust or inflated appearing. Hind femur enlarged with basal end bi-lobed. Hind tibia with 7 or 8 pairs of spines, but typically 8. Tympanum present, appearing as an opaque whitish disk. Abdomen cylindrical with distal portion distinctly, but not greatly enlarged in males. ***Terminalia of the male*** with short furcula that are widely separated at their bases (Fig. 8A, B). Supra-anal plate (Fig. 8A, B) broadly triangular, being broader than long, median grove notably indistinct. (Fig. 8A). Cercus of the male (Figs 3G, 8A, B) triangular, being longer than wide, acutely pointed distally. Subgenital plate with a low, but even dorsal margin, and a distinct median carina (Fig. 8A).

**Figure 4.**
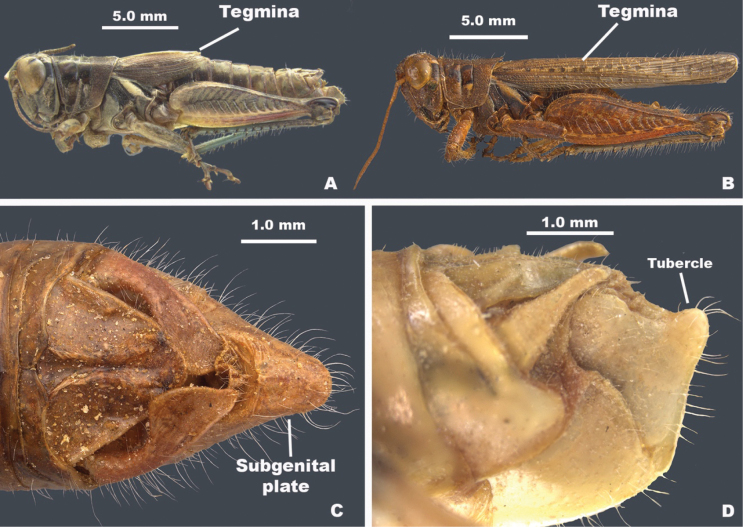
**A** brachypterous *Melanoplus***B** macropterous *Melanoplus***C** terminalia of *Barytettix* showing the conical subgenital plate **D** terminalia of *Aeoloplides* showing tubercle of the subgenital plate.

**Figure 5.**
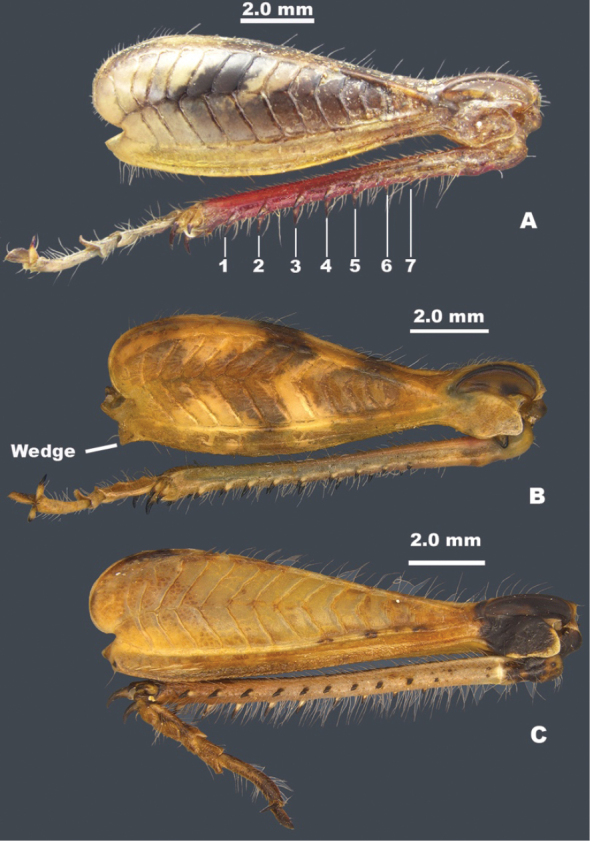
Hing legs **A***Oedomerus***B***Aeoloplides***C***Melanoplus*.

**Figure 6.**
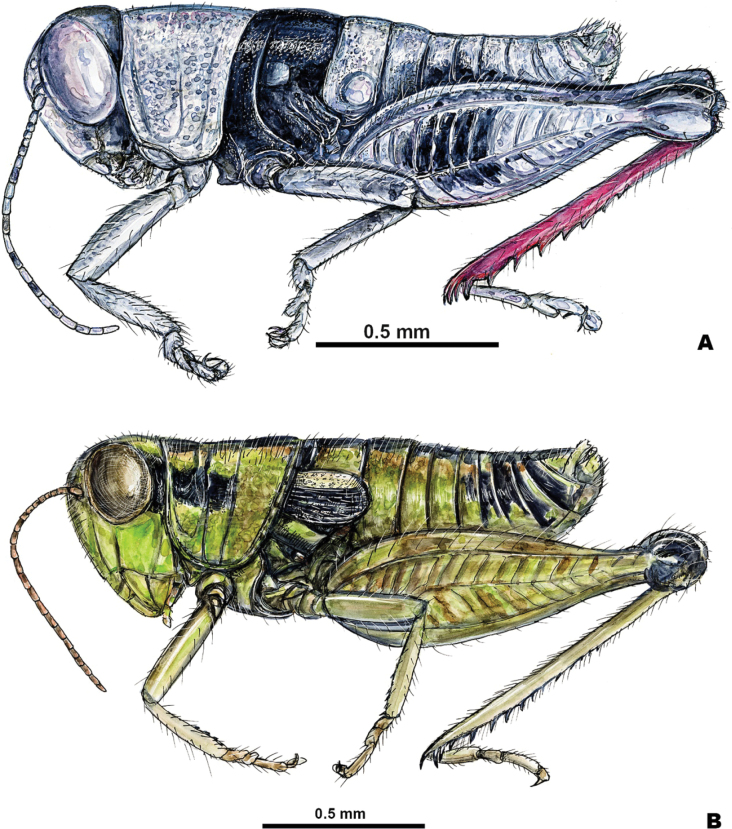
Males **A***Bajatettixcabopulmoensis***B***Ozmacrispeninsulae*. Produced by Joe MacGown.

**Figure 7.**
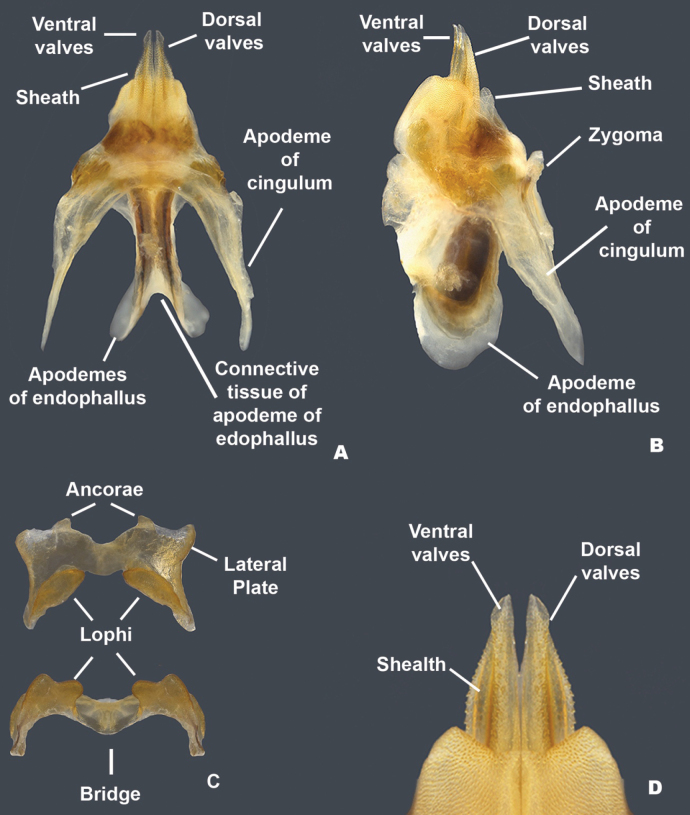
Morphology of the male phallic complex used in this work **A** dorsal view of the phallic complex **B** lateral view of the phallic complex **C** epiphallus dorsal and caudal views **D** caudal view of the aedeagus.

**Figure 8.**
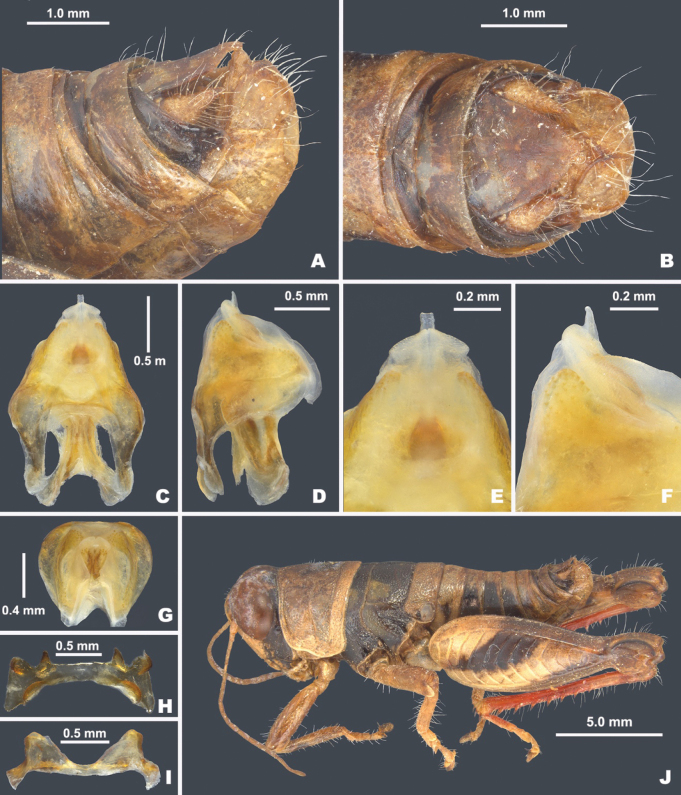
*Bajatettixcabopulmoensis***A** dorsal view of male terminalia **B** lateral view of male terminalia **C** dorsal view of phallic complex **D** lateral view of phallic complex **E** dorsal view of aedeagus **F** lateral view of aedeagus **G** caudal view of the aedeagus **H** dorsal view of epiphallus **I** caudal view of epiphallus **J** habitus.

***Phallic structures*.** The dorsal valves of the aedeagus are produced as broadly rounded plates in lateral view and in dorsal view are slanted caudally approximately 30 degrees medially to distally. The dorsal valves are widest in the middle and taper more sharply toward the distal end than at the base (Fig. 8C, E). In dorsal view, are parallel and join medially to form a quadrate process that extends slightly beyond the dorsal valves. In lateral view, the ventral valves appear as blunt tipped acutely angled triangle (Fig. 8D, F). In caudal view, the dorsal valves are broad arches, and the ventral valves meet medially to form a concave channel (Fig. 8G). Rami of the cingulum expanded into a broad plate in lateral view (Fig. 8D, F). Zygoma obsolete. The epiphallus is of the typical melanoploid shape, having lophi, ancorae, and an undivided bridge (Fig. 8H, I). More precisely, the epiphallus of *Bajatettix* have a slightly concave bridge, acutely sloping lophi with a broadly rounded apex, convexly curved lateral plates that are subdeltate in shape with a rounded anterior lobe and a rounded caudal tip, and ancora that are triangular, often tapering to a point (Fig. 8H, I). See Fig. 7 for labeled image.

**Figure 9.**
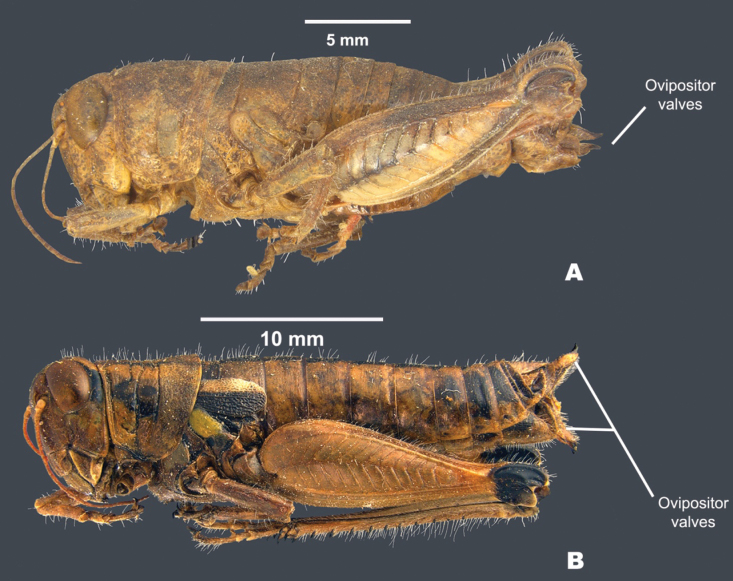
Female habitus of showing ovipositor valves **A***Bajatettixcabopulmoensis***B***Ozmacrispeninsulae*.

**Females** are similar to the males, but differ in being larger, more robust, and in the shape of the terminalia (Fig. 9A). ***Terminalia of female*** with triangular cerci and ovipositor valves that are subequal in length. The dorsum of the dorsal valves is nodose to slightly serrate proximally and concave and upcurving to a tip distally; the ventral valves have ventral margins curving basally and then about mid-point abruptly straighten distally (Fig. 9A).

**Figure 10.**
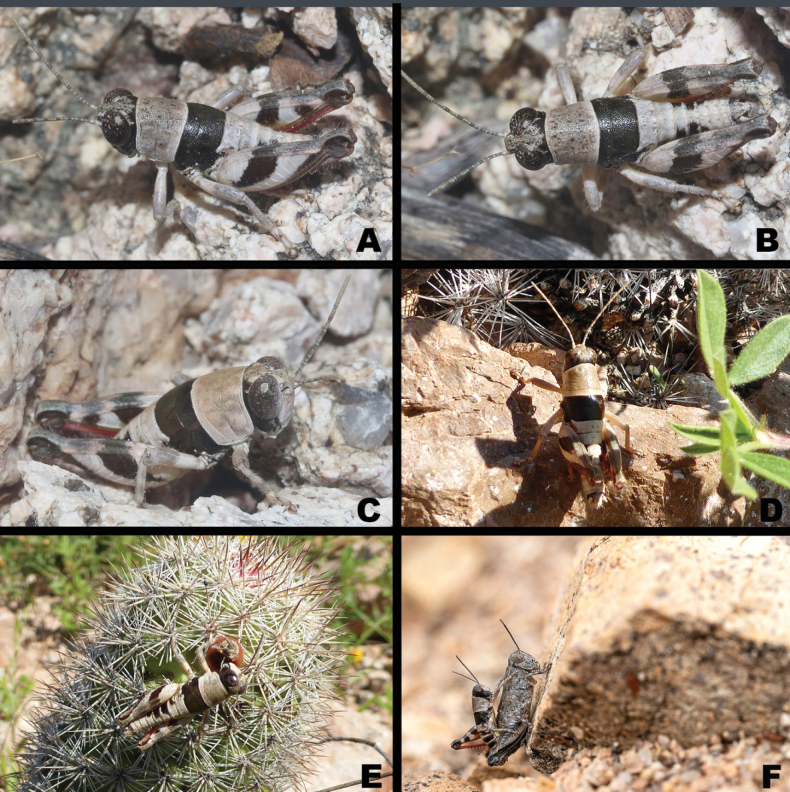
*Bajatettix* images from iNaturalist **A** male is lateral view **B** male in dorsal view **C** male in semi-anterior view **D** male in dorsal view **E** male feeding on cactus fruit **F** pair in copula Photo credit: James Baily (**A–C**), Jim Roberts (**D, E**), Michael Schmidt (**F**).

***Coloration*** overall ecru (grayish yellow) with raw umber (dark brown) bands on the head, mesothorax, abdomen, and hind femur (Figs 6A, 8J, 10A–F). Antenna ecru. Head ecru with a raw umber stripe on the gena. Pronotum with prozona ecru without a post ocular stripe; Mesothorax cinereous raw umber; metathorax ecru. The fore and middle legs ecru and unmarked. Hind femur ecru with an oblique cinereous raw umber band laterally that continues onto the dorsum; a dark crescent at the upper lateral femorotibial joint; hind femur coral red with black tipped spines. Abdomen ecru with a lateral raw umber stripe on some segments, especially in line with the oblique band on the hind femur.

##### Diagnosis.

*Bajatettix* lacks wings which easily differentiates it and *Psilotettix* from *Oedomeris* and other melanoplines on the Baja peninsula. The body surface of *Bajatettix* is generally smooth with the mesothorax and metathorax covered with a broad dark band (Figs 2B, 6A), whereas the surface of *Psilotettix* is rugose and punctate and variously colored.

##### Type species.

*Bajatettixcabopulmoensis* sp. nov.

##### Etymology.

Prefix “*Baja*- “from Baja California, where the genus is endemic, and the suffix “-*tettix*” (Greek) meaning grasshopper.

##### Suggested common name.

Saltadorito from the Spanish meaning tiny leaper.

#### ﻿ Bajatettix cabopulmoensis sp. nov.

Taxon classificationAnimaliaOrthopteraAcrididae

E959021D-6330-5E48-B0D5-557001635156

https://zoobank.org/38DE9518-301A-46FE-984A-88F3A8687AEB

**Figure 11.**
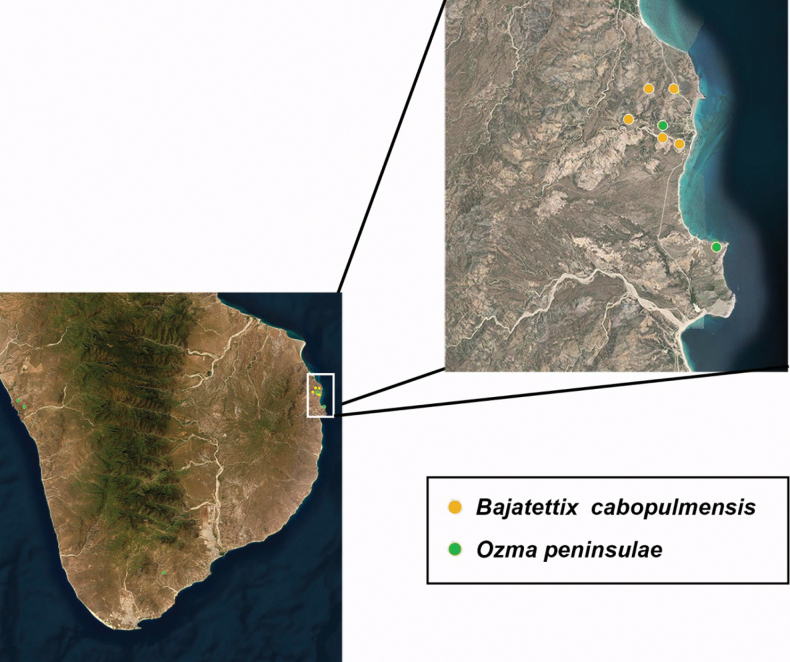
Distribution of *Bajatettixcabopulmoensis*.

Figs 2B , 3G , 6A , 8A–J , 9A , 10A–F , 11

##### Diagnosis.

*Bajatettixcabopulmoensis* is a medium-sized, wingless, gray grasshopper with broad dark-brown stripes on the head, thorax, and hind femurs that is endemic to the Baja peninsula (Figs 2B, 8J). The wingless state separates it from other melanoplines on the peninsula with the exception of members of *Psilotettix*, which it can be distinguished from by having a combination of a smooth body surface and the dark-brown markings on the head, thorax, and hind femur. While the genus is monotypic, the characters of the male terminalia and genitalia typically used for species-level identification in the Melanoplinae are detailed in the generic description above and illustrated in Fig. 8A–I.

##### Male measurements

**(mm).** (*n* = 7). Body length 17.2–19.3 (mean = 18.1); pronotum length 3.4–3.9 (mean = 3.6); hind femur length 9.2–10.0 (mean = 9.4); cerci length 0.5–0.7 (mean = 0.6); basal width of cercus 0.3–0.5 (mean = 0.2); mid width of cercus 0.2–0.3 (0.2); cerci apex width 0.1 (mean = 0.1).

##### Phallus measurements

**(mm).** (*n* = 1). Length 0.5; dorsal valve apex width 0.1; dorsal valve middle width 0.7; dorsal valve basal width 0.5, ventral valve apex width 0.2, ventral valve middle width 0.2, ventral valve basal width 0.2.

##### Female measurements

**(mm).** (*n* = 2). Body length 20.5–22.3 (mean = 21.4); pronotum length 4.5–5.1 (mean = 4.8); hind femur length 11.0–11.7 (mean = 11.4); dorsal ovipositor valve length 1.0–1.3 (mean = 1.2); ventral ovipositor valve length 1.0–1.3 (mean = 1.2).

##### Type materials.

***Holotype*.** Mexico • 1♂ Baja Calif Sur; Spring; 15.7 MI S La RIV[B]ERA at KM 41.7, 3; NOV 1985; COLL 7; R. E. LOVE. Deposited in the MEM.

***Paratypes*.** Mexico • 4♂, 2♀; same data as for holotype.

##### iNaturalist observation numbers.

197445998, 107386512, 62495147, 50030285, 36764494, 39120245.

##### Habitat.

Most likely sarcocaulescent shrubland.

##### Distribution.

*Bajatettixcabopulmoensis* is known only from the Capo Pulmo region of the eastern Cape Region of southern Baja California (Fig. 10).

##### Etymology.

Specific epithet derived from the Cabo Pulmo region where the species is apparently endemic to and the suffix “-ensis” (Latin) meaning “originating from” or “inhabiting”. This name reflects the localized nature of the species and hopefully draws attention to the importance of conservation in this region.

##### Suggested common name.

Capo Pulmo Saltadorito.

##### Note.

I emailed Robert Love to confirm the locality of the type series and he responded, “In answer to your question, ‘La Ribera’ is (I guess) the right spelling for the collection site you asked about as it is the local spelling. In a 1972 trip, I found a sign at the junction from route MX 1 that directed to the town of ‘La Rivera’ while local signs said ‘La Ribera’. As ‘v’ and ‘b’ are pronounced nearly identically and the letters sometimes confounded in local Spanish, I originally listed the town as ‘Rivera’, but changed it on most later trips. I don’t know why I reverted to the ‘v’ form on 1985 labels, as this one is La Ribera in my collections database. I made two collections there, 1985 and 1989.” (R.E. Love pers. com.)

#### ﻿ Ozmacris gen. nov.

Taxon classificationAnimaliaOrthopteraAcrididae

0DDD69A2-66FF-549C-814B-BDD690894EC3

https://zoobank.org/C94CBDB1-CEE2-4E68-8045-C451A0A1CFCE

Figs 2C , 3H , 6B , 9B , 12A–J , 13A–E , 14A–D , 15A–D , 16

##### Generic description.

**Figure 12.**
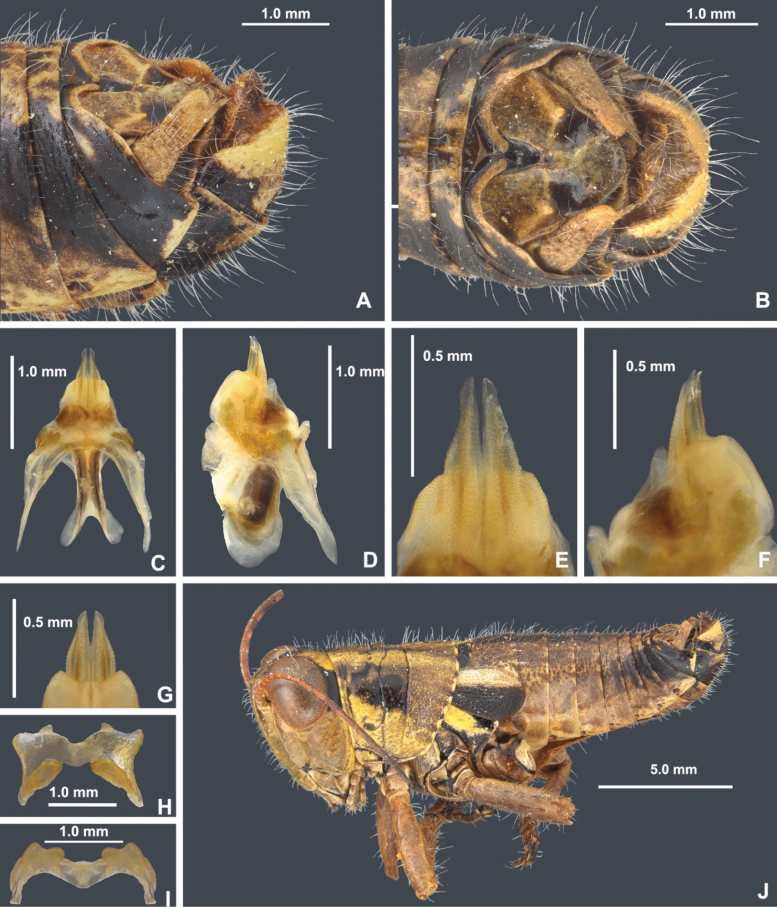
*Ozmacrispeninsulae***A** dorsal view of male terminalia **B** lateral view of male terminalia **C** dorsal view of phallic complex **D** lateral view of phallic complex **E** dorsal view of aedeagus **F** lateral view of aedeagus **G** caudal view of the aedeagus **H** dorsal view of epiphallus **I** caudal view of epiphallus **J** habitus.

***External morphology*.** A genus of small (15.0–23.5 mm), brachypterous grasshoppers (Figs 2C, 6B 12J). ***Head*** moderately sized and in equal in width to the anterior edge of the prozona; vertex between the eyes much wider than the basal antennomere; fastigium broad rounded, being more pronounced dorsally than ventrally, with a narrow deep medial depression dorsally, and a broad, shallow depression anteriorly. Eyes somewhat prominent, especially in males. Three ocelli present. Antennae filiform, usually with 22 flagellomeres in males, and 24–25 in females; nearly cylindrical, but slightly flattened dorso-ventrally; equal in width throughout, except two basal articles. Clypeus trapezoidal with lateral sulci and a shallow medial notch on the ventral edge. ***Thorax*** with prosternal spine well developed, broadly rounded distally. Pronotum convex in cross section, anterior margins sub-truncate, posterior margin broadly rounded, medial carina cut by three sulci, lateral carinae absent and humeral margins rounded. Prozona mostly smooth, but with light rugulation dorsally; lateral lobes broadly rounded (more so in females) with parallel lateral margins and the ventral margin sharply angled caudally. Metazona lighly punctate anteriorly and heavily punctate caudally, with humeral margins rounded and in dorsal view, slightly diverging posteriorly. Median carina low, but distinct throughout, except where the sulci cross it. Anterior, median, and posterior sulci are apparent, and all dissect the median carina and nearly reach the ventral margin of the lateral lobes. Lateral pronotal margins broadly rounded throughout. Interspace between mesosternal lobes nearly twice as long as broad. Tegmina elongate lobate with rounded apicies; dorsal margins broadly separated dorsally, strongly veined, and extending little past the anterior margin of the second abdominal tergite. Pro and meso thoracic legs not robust or inflated appearing. Hind femur enlarged with basal end bi-lobed. Hind tibia with 10 or 11 pairs of spines, but typically 10. Tympanum present under tegmina, appearing as an opaque whitish disk. ***Abdomen*** cylindrical with distal portion distinctly, but not greatly enlarged in males. ***Terminalia of the male*** without furcula (Fig. 12A, B). Supra-anal plate (Fig. 12A, B) broadly triangular, being broader than long, with the anterior margin distinctly bi-lobate; the median groove anteriorly distinct with elevated sides but only extending approximately over half the plate (Fig. 12A). A low carina divides the apical and caudal halves and terminates in mid-distal short lateral spinules. Cercus of the male (Figs 3H, 12A, B) subquadrate, but longer than wide, with a small tooth on the ventral apical margin. species, Subgenital plate with a low, but even margin.

**Figure 13.**
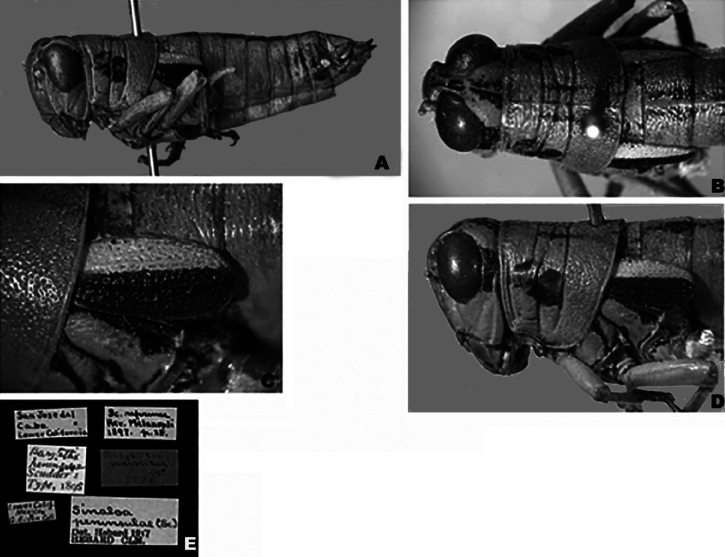
Type specimen images of *Ozmacrispeninsulae***A** lateral habitus view **B** dorsal view of head and thorax **C** tegmina **D** lateral view of head and thorax **E** type labels.

**Figure 14.**
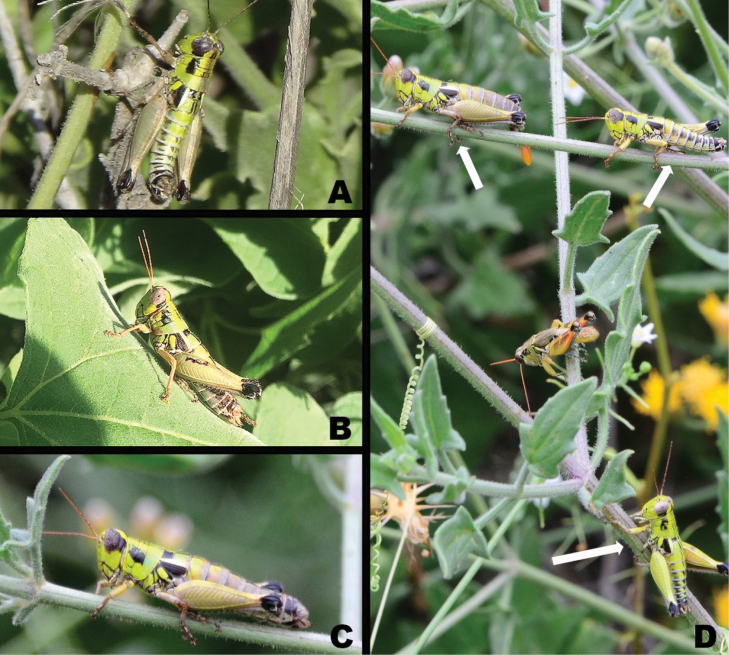
*Ozmacris* images from iNaturalist **A** male in dorsolateral view **B** female in lateral view **C** another female in lateral view **D** group image showing multiple individuals—white arrows indicate *Ozmacris* specimens while the central individual is likely part of the *Melanoplusaridus* group. Photo credits: Alvero San Jose Elizundia (**A**), Lauren Harter (**B**), Damon Tighe (**C, D**).

**Figure 15.**
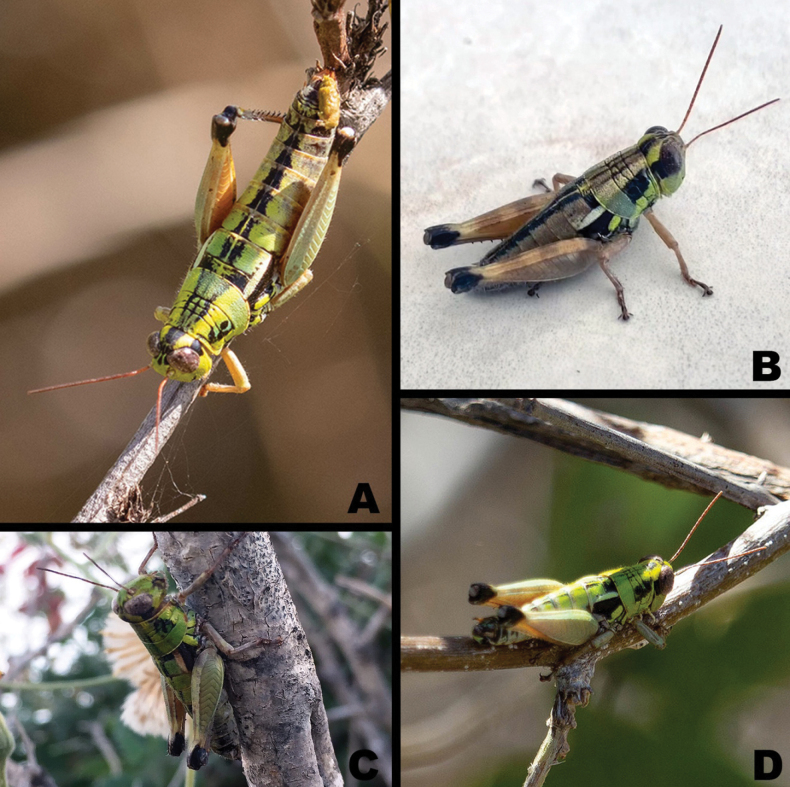
*Ozmacris* image form iNaturalist **A** female in dorsal view **B** female in dorso-lateral view **C** female in antero-dorsal view **D** male is dorso-lateral view. Photo credits: Dan Fitzgerald (**A**), Demente Villegas (**B**), Lauren Harter (**C**), Tania Perez Fiol (**D**).

**Figure 16.**
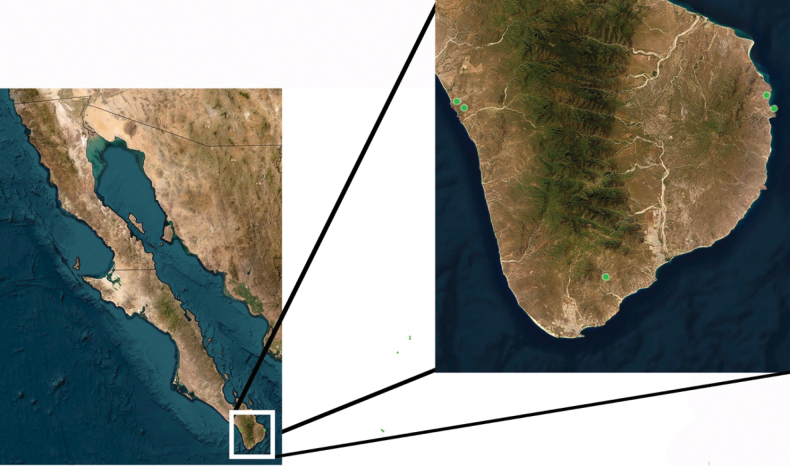
Distribution of *Ozmacrispeninsulae*.

***Phallic structures*.** The dorsal valves of the aedeagus are produced as cuneiform plates that are sculptured with small serrations and are slightly shorter than the ventral valves (Fig. 12C, E). The ventral valves are linear, parallel plates with distinct angles along the disto-lateral margins. They have a smoother texture and are slightly longer than the dorsal valves (Fig. 12C–G). The epiphallus is of the typical melanoploid shape, having lophi, ancorae, and an undivided bridge, but more precisely, *Ozmacris* has a concave bridge, broadly bidentate lophi, concavely curved lateral plates that are subdeltate in shape with an angular anterior lobe and caudal tip, and ancora that are triangular (Fig. 12H, I). See Fig. 7 for labeled image.

**Females** are similar to the males, but differ in being larger, more robust, and in the shape of the terminalia (Figs 9B, 13A–D, 14B–D, 15A–C). ***Terminalia of female*** with triangular cerci and ovipositor valves that are subequal in length. The dorsal valves with their dorsal margin nodose proximally and slightly serrate distally, and with the distal apices concave and upcurving to a tip. The ventral valves with their ventral margins straight basally and then arching distally (Fig. 9B).

***Coloration*** light citron (green with a yellow tinge) overall, with individual variation that can have extremities with a light tan hue (Figs 2C, 6B, 9B, 12J, 14A–D, 15A–D). Antenna light testaceous (dull brick-red). Head citron with black markings, including a dorsal more or less broken, black band which follows the sulcus of the fastigium and broadens caudally, and a broad post-ocular stripe. Genae citron. Pronotum citron with an olivaceous tinge; lateral lobes marked with the post-ocular stripe beginning just behind the anterior border of the prozona across the mesosoma and then disappearing on the metazoa; disk with median carinae and two subdorsal lines. Tegmina black on the lower two-thirds, above third white (3A,13A–E). The fore and middle legs unmarked. Hind femur pallid citron, the entire geniculation except most of the lower lobe black; hind tibia brownish citron to pale blue with black spines. Abdomen with a narrow medial black stripe; medial carinae citron or testaceous depending on the individual.

##### Diagnosis.

*Ozmacris* is a medium-sized, brachypterous green grasshopper with bicolored tegmina, which easily differentiates it from other melanoplines on the peninsula (Figs 2C, 6B). It is separated from *Barytettix* in having oval tegmina, toothed male cerci, a subgenital plate that is not conical, and a distinct overall aedeagus shape. *Ozmacris* differs from *Sinaloa* by having bi-colored tegmina, toothed male cerci, less developed furculae, and a distinct overall aedeagus shape.

##### Type species.

*Barytettixpeninsulae* Scudder, 1897 (by original designation).

##### Etymology.

*Ozmacris* is a combination of “Ozama” after Princess Ozma from L. Frank Baum’s *Ozma of Oz* (Baum 1907) and *acris* from the Greek word for grasshopper. The name is a reference to the scene where Princess Ozma is transformed by the Nome King into an emerald-green grasshopper ornament beneath the Deadly Desert. Baum (1907: 180) described the scene: “The room was quite empty of life after that. The Nome King had gained a new ornament, for upon the edge of the table rested a pretty grasshopper, seemingly crafted from a single emerald. It was all that remained of Ozma of Oz”.

##### Suggested common name.

I designate the word ‘bauble’ as the common name for this genus of grasshopper. This name evokes the image of the grasshopper as a shiny, jewel-like object or trinket.

#### ﻿ Ozmacris peninsulae

Taxon classificationAnimaliaOrthopteraAcrididae

(Scudder) comb. nov.

53EEEDC0-B9D9-56CB-B6B7-EC18117FF7BC

Figs 2C , 3H , 6B , 9B , 12A–J , 13A–E , 14A–D , 15A–D , 16

Barytettix peninsulae Scudder, 1897: 28; Rehn and Hebard 1912: 74; Otte and Cohn 2002.
Sinaloa peninsulae (Scudder): Bruner 1908: 305; Hebard 1925: 288; Otte 1995: 413.

##### Diagnosis.

*Ozmacrispeninsulae* is a medium-sized, brachypterous, greenish grasshopper with bicolored (black and white) oval tegmina (Figs 2C, 6B). The green coloration and shape and color of the tegmina separate it from most other melanoplines on the Baja peninsula. It can be further distinguished from *Barytettix* in having a non-conical subgenital plate and toothed male cerci. While the genus is monotypic, the characters of the male terminalia and genitalia typically used for species-level identification in the Melanoplinae are detailed in the generic description above and illustrated in Fig. 12A–I.

##### Male measurements

**(mm).** (*n* = 1). Body length 15.0; pronotum length 4.1; tegmen length 2.1; hind femur length 11.6; cerci length 0.9; basal width of cercus 0.3; mid-cercal width 0.3; cerci apex width 0.3.

##### Phallus measurements

**(mm).** (*n* = 1). Length 0.6; apex width 0.2; middle width 0.5; basal width 0.5. Because both pairs of valves are largely parallel and fit closely together the structures were measured as a single unit.

##### Female measurements

**(mm).** (*n* = 3). Body length 18.6–23.5 (mean = 21.2); pronotum length 4.2–5.0 (mean = 4.6) tegmen length 2.5–3.2 (mean = 2.9); hind femur length 10.5–13.2 (mean = 11.8); dorsal ovipositor valve length 1.7–2.0 (mean = 1.7); ventral ovipositor valve length 1.3–1.7 (mean = 1.7).

##### Holotype.

Lower California • 1♀; G. Eisen Deposited in the California Academy of Science.

##### Specimens examined.

Mexico • **Baja California Sur**, 1♀; Cabo San Lucas; 17 October 1974 • 1♂,1♀; 3 km S Todos Santos at Km 55.5; 28 August 1995; el 120 m; DB Weissman, DC Lightfoot (DB Weissman Stop # 95–89) • 1♀ 11.2 km S Todos Santos on road to Todos Santos; 27 September 1979, DB Weissman, DC Lightfoot; #79–208.

##### iNaturalist observation numbers.

252092963, 191705108, 188043064, 99096505, 98953983, 17968068, 19187122.

##### Habitat.

From personal communication with Dave Weissman: “At S95–89, we were specifically looking for *B.peninsulae*. The GPS for the near exact spot is 23.418251 −110.214600, 23 m elevation. The vegetation was scrub thorn but heavily overgrazed. The area had had adequate rain by the vegetation. We searched for 1 hour looking for hoppers. We checked many plants and stomped a lot of bushes. The last instar male and female that we collected were both in bushes, most of which had few leaves. They were a bitch to catch when we saw them and my notes say that we got both that we saw. Nothing on the ground. I had my colleague Bruce Bartholomew (CAS Dept of Botany) ID some branches that I brought back: Ruelliacalifornica×peninsularis (Acanthaceae) was his tentative ID but made difficult since I am not sure if there were even leaves present.”

##### Distribution.

*Ozmacrispeninsulae* is known only from the Cape Region of Baja California (Fig. 16).

##### Suggested Common Name.

Baja bauble.

## ﻿Discussion

The two new grasshopper genera described here both appear to be endemic to the Cape Region of Baja California, a biogeographic province located at the southern tip of the peninsula. This region’s endemism is due to its combination of prolonged geographic isolation, diverse microhabitats, and climatic variability, which have collectively driven the evolution of unique species found nowhere else. Renowned for its unique biota, the Cape Region is characterized by high endemism and ecological diversity, shaped by prolonged geologic isolation, topographic heterogeneity, and varied weather conditions (Johnson and Ward 2002). This area harbors a variety of rare ecosystems, including xeric scrub in the lowlands, tropical dry forests on the lower slopes of the Sierra de La Laguna, and pine–oak forests at higher elevations (Hugo and Ezcurra 2007).

Phylogenetic evidence from various taxa suggests that the Baja peninsula experienced multiple submersions and uplifts during the Plio-Pleistocene, fragmenting it into islands. These events created temporary transpeninsular seaways that connected the Pacific Ocean with the Gulf of California (Murphy 1983; Riddle et al. 2000). Around 3 million years ago, during the late Tertiary, the Cape Region was an island, separated from the rest of the peninsula by the La Paz seaway, which is now represented by a low isthmus of marine deposits (Hausback 1984; Murphy and Aguirre-Leon 2002. Dolby et al. 2015). By the early Pleistocene, the peninsula had reached its current configuration. The Cape Region, which likely originated farther south near the present-day State of Jalisco, Mexico (Gastil and Jensky 1973), eventually joined the rest of the peninsula (Durham and Allison 1960; Brown et al. 1992).

*Bajatettix* and *Ozmacris* likely originated in the insular Cape Region during its period of isolation prior to the closure of the La Paz seaway. Pleistocene glacial cycles are hypothesized to have driven similar evolutionary processes in brachypterous North American melanopline grasshoppers. These cycles altered river systems, reshaped mountain ecosystems, and created isolated islands or sand ridges, leading to repeated patterns of population contraction, isolation, divergence, expansion, and secondary contact. Such dynamics facilitated species-level diversification in several *Melanoplus* groups (Knowles 2007; Woller 2017; Huang 2020). By the early Pleistocene, when the Cape Region reconnected with the rest of the peninsula, *Bajatettix* and *Ozmacris*—possibly due to habitat preferences or physiological adaptations to warmer Neotropical climates were restricted to the Cape Region. This pattern of endemism has also been observed in other taxa native to the Cape, such as butterflies (Brown et al. 1992).

This hypothesis requires testing with genomic data, which is currently lacking. Perhaps the key and descriptions presented here that will aid identification, will stir interest in the origins of the grasshopper fauna of Baja California, and contribute to the biogeographic story of this interesting region.

## Supplementary Material

XML Treatment for Bajatettix

XML Treatment for Bajatettix cabopulmoensis

XML Treatment for Ozmacris

XML Treatment for Ozmacris peninsulae

## ﻿Additional information

### Author ORCIDs

JoVonn G. Hill https://orcid.org/0000-0002-1892-7117

### Data availability

All of the data that support the findings of this study are available in the main text.
